# Taspase1 orchestrates fetal liver hematopoietic stem cell and vertebrae fates by cleaving TFIIA

**DOI:** 10.1172/jci.insight.149382

**Published:** 2021-08-09

**Authors:** Hidetaka Niizuma, Adam C. Searleman, Shugaku Takeda, Scott A. Armstrong, Christopher Y. Park, Emily H. Cheng, James J. Hsieh

**Affiliations:** 1 Human Oncology & Pathogenesis Program, Memorial Sloan Kettering Cancer Center, New York, New York, USA.; 2 Department of Pediatrics, Tohoku University School of Medicine, Sendai, Japan.; 3 Department of Medicine, Washington University School of Medicine, St. Louis, Missouri, USA.; 4 Department of Pediatric Oncology, Dana-Farber Cancer Institute, Boston, Massachusetts, USA.; 5 Department of Pathology, NYU School of Medicine, New York, New York, USA.; 6 Department of Pathology, Memorial Sloan Kettering Cancer Center, New York, New York, USA.

**Keywords:** Development, Stem cells, Embryonic development, Genetic diseases, Stem cell transplantation

## Abstract

Taspase1, a highly conserved threonine protease encoded by *TASP1*, cleaves nuclear histone-modifying factors and basal transcription regulators to orchestrate diverse transcription programs. Hereditary loss-of-function mutation of *TASP1* has recently been reported in humans as resulting in an anomaly complex syndrome, which manifests with hematological, facial, and skeletal abnormalities. Here, we demonstrate that Taspase1-mediated cleavage of TFIIA**α**-**β**, rather than of MLL1 or MLL2, in mouse embryos was required for proper fetal liver hematopoiesis and correct segmental identities of the axial skeleton. Homozygous genetic deletion of Taspase1 disrupted embryonic hematopoietic stem cell self-renewal and quiescence states and axial skeleton fates. Strikingly, mice carrying knockin noncleavable mutations of TFIIA**α**-**β**, a well-characterized basal transcription factor, displayed more pronounced fetal liver and axial skeleton defects than those with noncleavable MLL1 and MLL2, 2 trithorax group histone H3 trimethyl transferases. Our study offers molecular insights into a syndrome in humans that results from loss of TASP1 and describes an unexpected role of TFIIA**α**-**β** cleavage in embryonic cell fate decisions.

## Graphical abstract

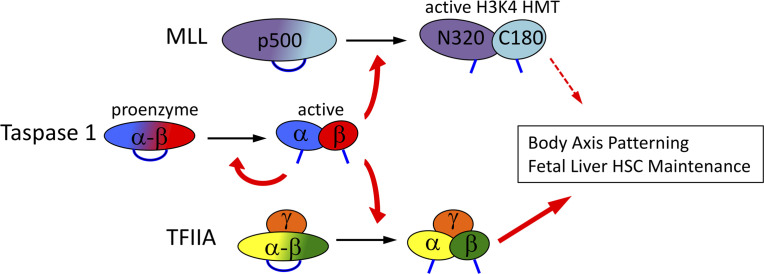

## Introduction

Recently, a novel human hereditary anomaly syndrome was recognized in association with loss-of-function mutations in the *TASP1* gene (1–3). Those patients were characterized with microcephaly, developmental delay, distinctive facial features, and other anomalies, including anemia, thrombocytopenia, and lymphocytopenia (2, 3). *TASP1* codes for Taspase1, which is an evolutionarily conserved threonine protease that cleaves and regulates nuclear proteins, most notably, MLL (KMT2A, also known as MLL1) and TFIIAα-β (4–6). Taspase1 is a 50 kD α-β proenzyme that undergoes intramolecular autoproteolysis to produce mature, active α28/β22 heterodimeric protease (4, 6–8). Cleavage of Taspase1 substrates occurs distal to the aspartate residue within the conserved IXQL(V)D/G motif (4, 9).

The bona fide Taspase1 substrates are MLL1, MLL2 (also known as KMT2B), TFIIAα-β (also known as GTF2A1), ALF (TFIIA-like factor, also known as GTF2A1L) and *Drosophila* HCF (dHCF) (4, 5, 10–12). MLL1, a member of the trithorax group (Trx-G) epigenetic modifiers, is a histone methyl transferase (HMT) that trimethylates H3 at lysine 4, generating a histone mark of active transcription (13, 14). In the absence of proteolytic activation, immature MLL1 polypeptide displays reduced HMT activity and, hence, functions as a hypomorphic mutant (11). TFIIA, comprising 3 polypeptides (α, β, and γ), complexes with TATA-binding protein (TBP) and constitutes an integral part of the basal transcription machinery (15–19). Precursor TFIIAα-β, encoded by a single gene, *Gtf2a1*, is processed into TFIIAα and -β subunits by Taspase1-mediated cleavage (5). Complexes of TFIIAα-β/γ containing noncleaved TFIIAα-β have transcriptional activity in vitro, and its maturation into TFIIAα/β/γ increases its susceptibility to regulatory degradation (5). In addition, TFIIAα-β/γ and TFIIAα/β/γ are different in the pattern of interactions with TFIID, an important factor for promoter recognition (20). Since TBP-like protein was reported to be a negative regulator of the Taspase1-mediated processing of TFIIA, Taspase1 may fine-tune the transcription of genes through these factors (21). Previous studies revealed that the proteolytic cleavage of TFIIA is critical for male spermiogenesis (22) and craniofacial development (23).

Taspase1-mediated cleavage of each substrate may account for various phenotypes of Taspase1 deficiency, or, alternatively, protease-independent biological activities of Taspase1 may exist. Here, we performed mouse genetic studies to interrogate the biological significance of cleavage of MLL1, MLL2, and TFIIA. Taspase1-mediated cleavage of TFIIA has the most prominent effects on fetal hematopoiesis as well as on the specification of axial skeleton, offering molecular insights concerning a syndrome in humans that results from loss of TASP1.

## Results

**Figure 1:**
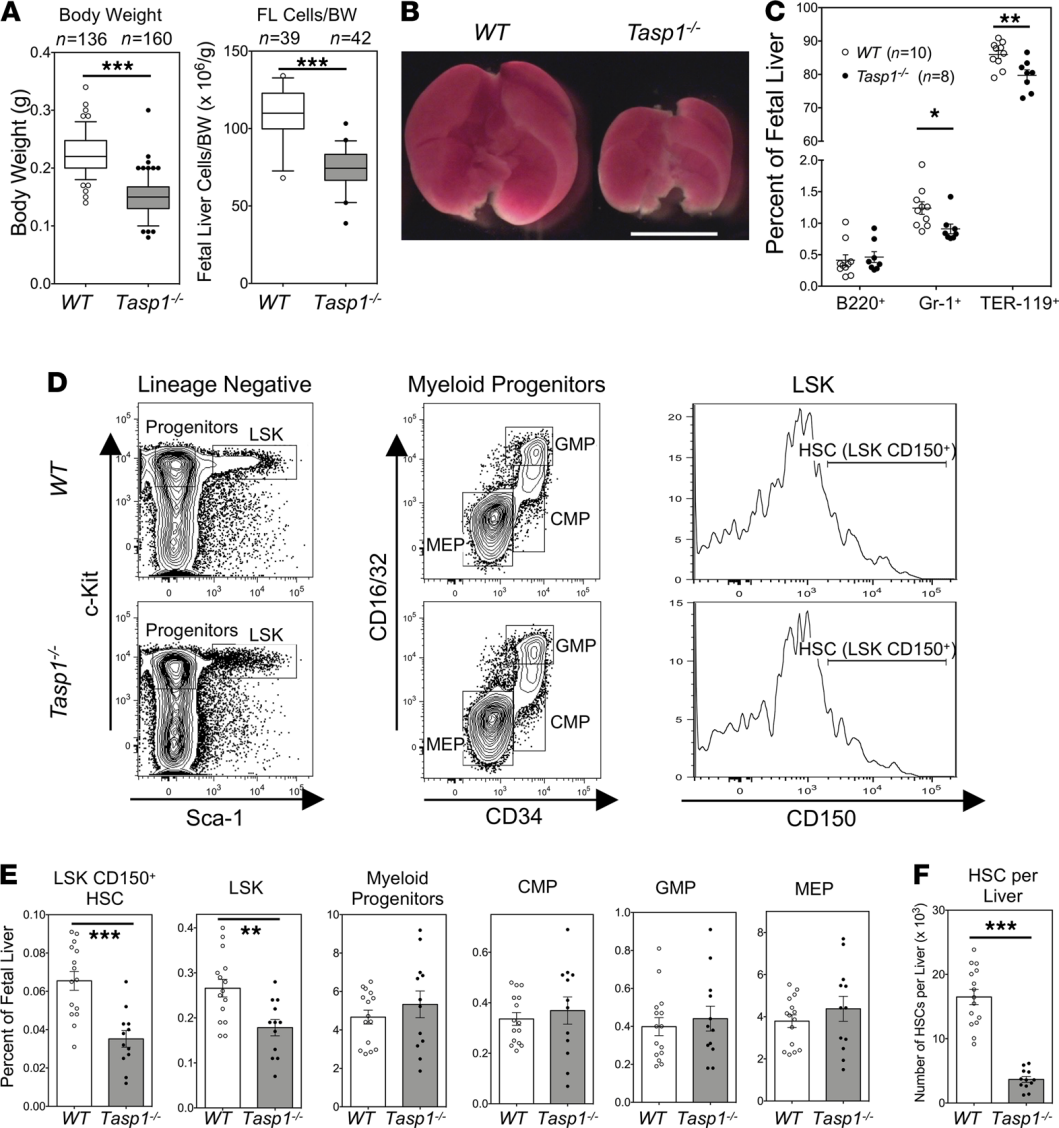
Taspase1 deficiency results in fetal liver hematopoietic stem cell defects. (**A**) Body weight and cell numbers of fetal livers (FLs) normalized to body weight of E14.5 embryos of the indicated WT and Taspase1-knockout (*Tasp1^–/–^*) embryos. Boxes contain the 25th to 75th percentiles of data sets, with 50th percentile center lines, and whiskers mark the 5th and 95th percentiles. Outliers are shown by dots. (**B**) Images of E14.5 FLs of the indicated genotypes. Scale bar: 2 mm. (**C**) Frequency of E14.5 FL cells committed to erythroid (TER-119^+^), myeloid (Gr-1^+^), and B cell (B220^+^) lineages. (**D**) Progenitor and stem cell analyses of E14.5 FL cells by flow cytometry. Lin^–^Sca-1^+^c-Kit^+^ cells are defined as LSK, and Lin^–^Sca-1^–^c-Kit^+^ cells as myeloid progenitors. Myeloid progenitors are subdivided into CMP, GMP, and MEP by CD34 and CD16/32. LSK CD150^+^ cells are defined as HSCs. (**E**) Quantification of stem and progenitor cells of 15 WT and 12 *Tasp1^–/–^* FLs. (**F**) HSCs per E14.5 FL of the indicated genotypes. Numbers were calculated by multiplying FL cellularity and HSC frequency. Data are shown as the mean ± SEM. *P < 0.05, **P < 0.01, ***P < 0.001 by Mann-Whitney *U* test. HSC, hematopoietic stem cell; CMP, common myeloid progenitor; GMP, granulocyte-monocyte progenitor; MEP, megakaryocyte-erythrocyte progenitor.****

To investigate the physiological function of Taspase1, we generated *Tasp1*-deficient mice (*Tasp1^–/–^* mice). Our initial report demonstrated that these animals displayed marked homeotic transformation of the axial skeleton and a decrease in overall body size (11). Furthermore, *Tasp1^–/–^* mice die shortly after birth, partly due to feeding defects (11). Here, we further examined the role of Taspase1 in embryogenesis and initiated studies by comparing these *Tasp1^–/–^* and WT littermate embryos. *Tasp1^–/–^* E14.5 embryos show a severe developmental retardation, reflected by an approximately 40% reduction in overall weight in comparison to that of WT embryos (Figure 1A). Furthermore, *Tasp1^–/–^* embryos have markedly smaller fetal livers (Figure 1B). The decrease in fetal liver cell number was significant even after normalizing for reduced body weight (*P* < 0.001) (Figure 1A).

We next analyzed the fetal liver for mature blood cell lineages and the hematopoietic stem and progenitor cell compartments using multicolor flow cytometry. The analysis of E14.5 fetal livers showed that *Tasp1^–/–^* and WT fetal livers had little difference in the populations of B220^+^ B cells, Gr-1^+^ granulocytes, and TER-119^+^ red blood cells (Figure 1C). In contrast, the stem and progenitor cell populations, marked by Lin^–^Sca-1^+^c-Kit^+^ staining (so-called LSK cells), were reduced in *Tasp1^–/–^* fetal livers (Figure 1, D and E). LSK cells can be further specified as multipotent progenitor cells (MPPs; Lin^–^Sca-1^+^c-Kit^+^CD150^–^) or hematopoietic stem cells (HSCs; Lin^–^Sca-1^+^c-Kit^+^CD150^+^) (24, 25). In *Tasp1^–/–^* fetal livers, the abundance of HSCs was found to be reduced to approximately half that of the WT fetal liver (Figure 1E), resulting in an approximately 78% decrease in the absolute number of HSCs (Figure 1F). No difference in the frequency of Lin^–^Sca-1^–^c-Kit^+^ myeloid progenitors was observed, whereas absolute numbers of these cells were decreased. Specifically, in *Tasp1^–/–^* fetal livers, the relative abundance of common myeloid progenitors (CMPs), granulocyte-monocyte progenitors (GMPs), and megakaryocyte-erythrocyte progenitors (MEPs) was normal (Figure 1, D and E). Together, our data indicate that, in the mouse fetal liver, Taspase1 may be required for the development and maintenance of the HSC compartment.

**Figure 2:**
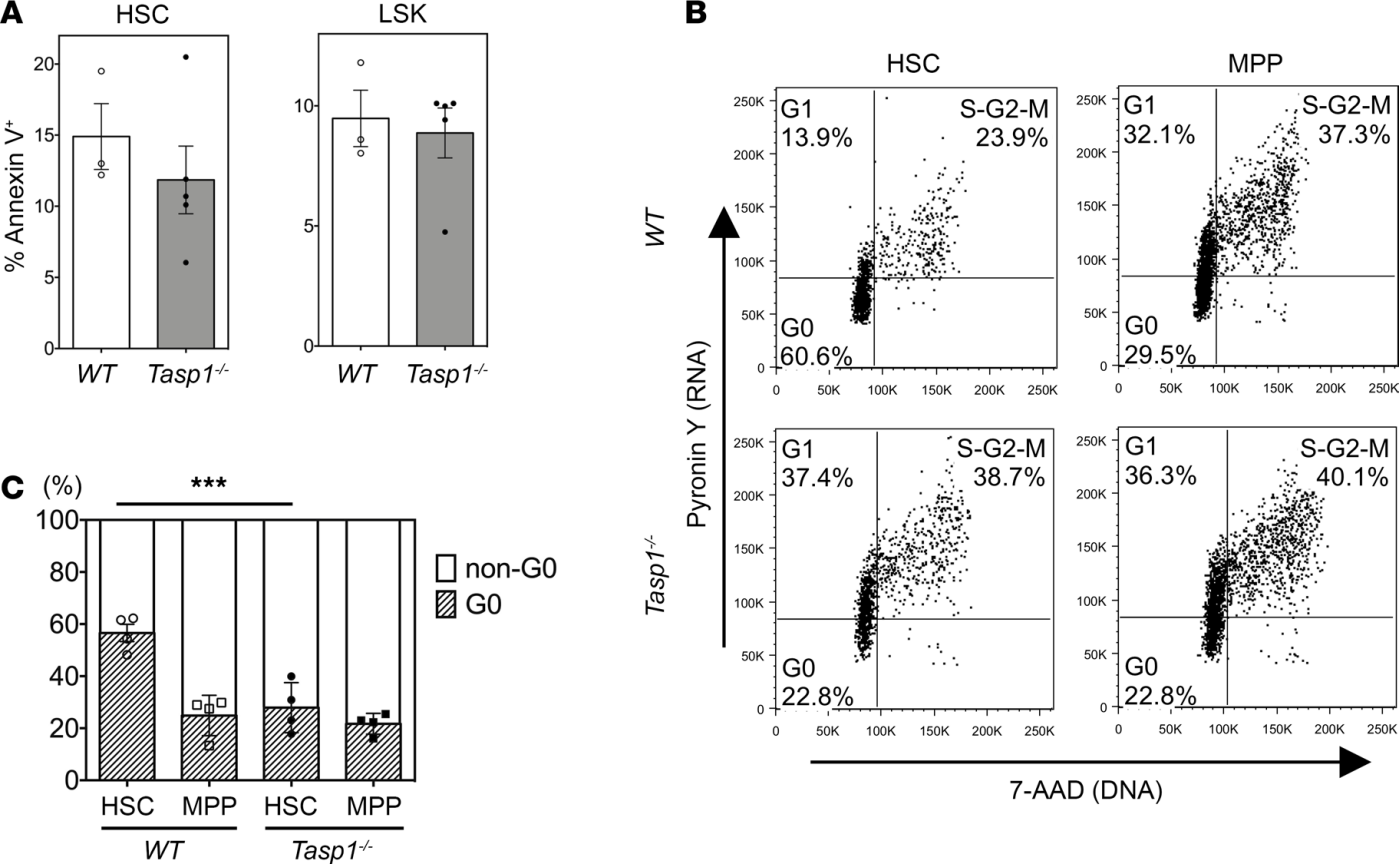
Tasp1–/– fetal liver hematopoietic stem cells exhibit an aberrant cell cycle profile reminiscent of multipotent progenitor cells. (**A**) Cell death analysis quantified by annexin V staining. The frequency of annexin V^+^ cells in fetal liver (FL) hematopoietic stem cell (HSC) and LSK cells of the indicated genotypes was displayed (WT, *n* = 3; *Tasp1^–/–^*, *n* = 5). Data are shown as the mean ± SEM. (**B** and **C**) Cell cycle analysis of FL HSCs and multipotent progenitor cells (MPPs). Cells were stained with 7-AAD and pyronin Y to assess DNA and RNA contents, respectively. (**B**) The quadrant gates of the representative plots based on DNA and RNA contents are outlined as G_0_, G_1_ and S/G_2_/M phases. (**C**) The frequency of HSCs and MPPs in G_0_ and non-G_0_ (G_1_ and S/G_2_/M) phases. Data are shown as the mean ± SEM (*n* = 4 for all samples). *P* values of 4 independent experiments by χ^2^ testing were combined by the Fisher’s method; ***P < 0.001.

Accordingly, we investigated how Taspase1 loss leads to impaired fetal liver HSC renewal. Loss of Taspase1 activity could lead to increased cell death and/or aberrant cell cycle control. Cell death analysis of LSK cells and HSCs showed no increase of apoptosis in *Tasp1^–/–^* fetal livers (Figure 2A). On the other hand, cell cycle analysis of MPPs and HSCs yielded a marked difference between WT and *Tasp1^–/–^* cells. Most notably staining of DNA and RNA with 7-AAD and pyronin Y, respectively, showed that more *Tasp1^–/–^* fetal liver HSCs exited stem cell quiescence, with decreased number of cells in the G_0_ phase, whereas the majority of WT HSCs were in the G_0_ phase, with fewer cells at G_1_ and S/G_2_/M (Figure 2, B and C). In contrast, *Tasp1^–/–^* fetal liver MPPs displayed similar cell cycle profile as WT MPPs. Strikingly, the cell cycle profile observed of *Tasp1^–/–^* fetal liver HSCs resembles that of MPPs (Figure 2B).

**Figure 3:**
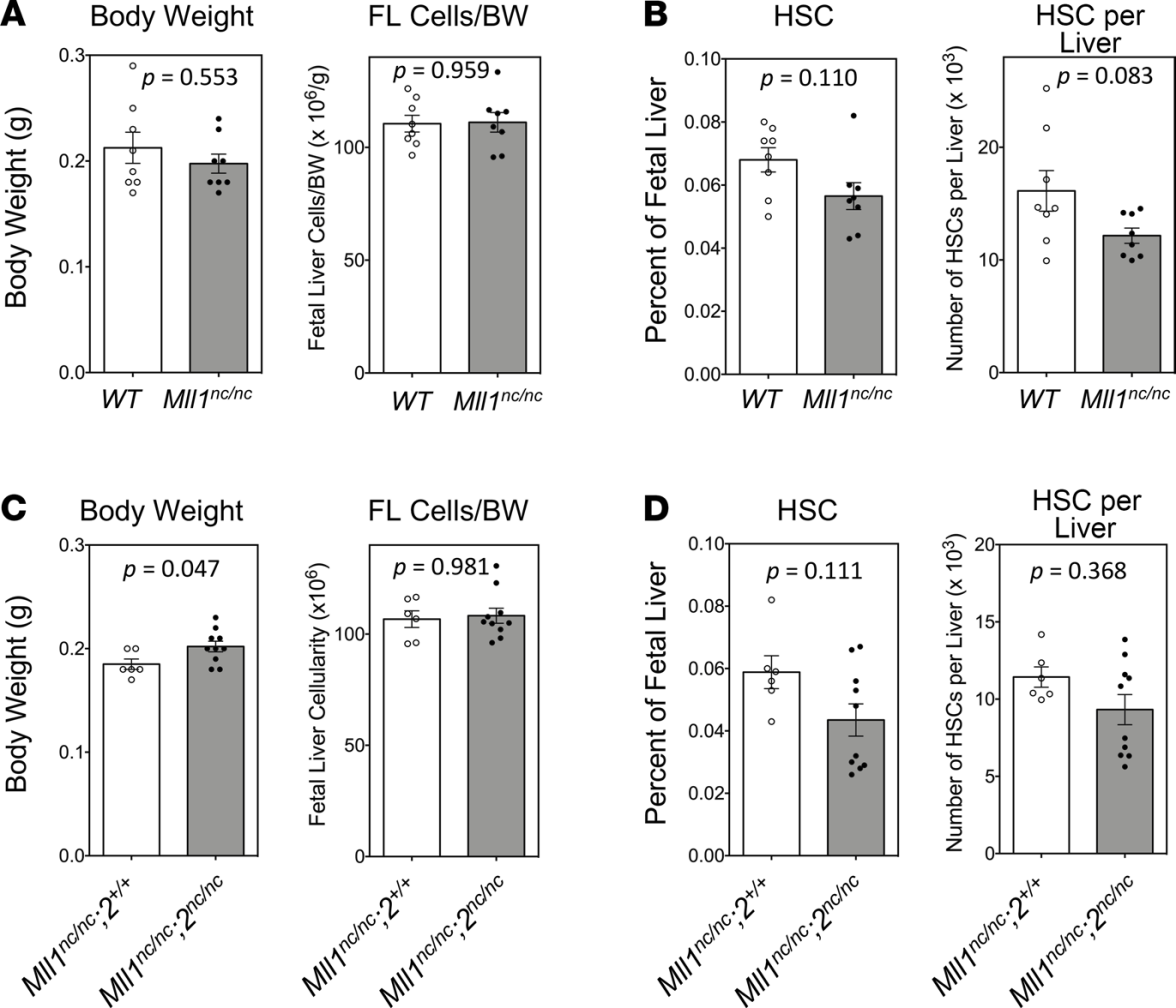
Hematopoietic stem cell quantification of Mll1nc/nc and Mll1nc/nc;2nc/nc fetal livers. (**A** and **B**) Embryos obtained by intercrossing *Mll1^nc/+^* mice were analyzed. (**A**) Body weight and normalized E14.5 fetal liver (FL) cellularity. (**B**) Frequency of hematopoietic stem cells (HSCs) and number of HSCs per FL (WT, *n* = 8; *Mll1^nc/nc^*, *n* = 8). (**C** and **D**) Embryos obtained by intercrossing *Mll1^nc/nc^*;*2^nc/+^* mice were analyzed. (**C**) Body weight and normalized E14.5 FL cellularity. (**D**) Frequency of HSCs and number of HSCs per FL (*Mll1^nc/nc^*;*2^+/+^*, *n* = 6; *Mll1^nc/nc^*;*2^nc/nc^*, *n* = 10). Data are shown as the mean ± SEM. *P* values were determined by Mann-Whitney *U* test.

To elucidate by which substrate(s) cleavage of Taspase1 regulates fetal liver hematopoiesis, we first focused on the MLL family proteins MLL1 and MLL2. MLL1 is the best-characterized Taspase1 substrate, controls *Hox* and *Cylclin* gene expression, and plays a critical role in both fetal and adult hematopoiesis (26–28). Homozygous knockin of noncleavable (nc) *Mll1* mutant alleles (*Mll1^nc/nc^*) at the endogenous *Mll1* locus did not reduce the overall fetal liver cellularity (Figure 3A) but incurred a minor decrease in frequency and number of HSCs (Figure 3B). As published data suggest a partial redundancy between Mll1 and Mll2 (11), we analyzed E14.5 fetal livers of *Mll1^nc/nc^*;*2^nc/nc^* embryos bearing homozygous knockin of nc *Mll1* and nc *Mll2* mutant alleles at their native genomic loci and did not detect reduced fetal liver cellularity or body weight (Figure 3C). Similarly, the reduced abundance or absolute number of HSCs in *Mll1^nc/nc^;2^nc/nc^* fetal livers was not statistically significant (Figure 3D).

**Figure 4:**
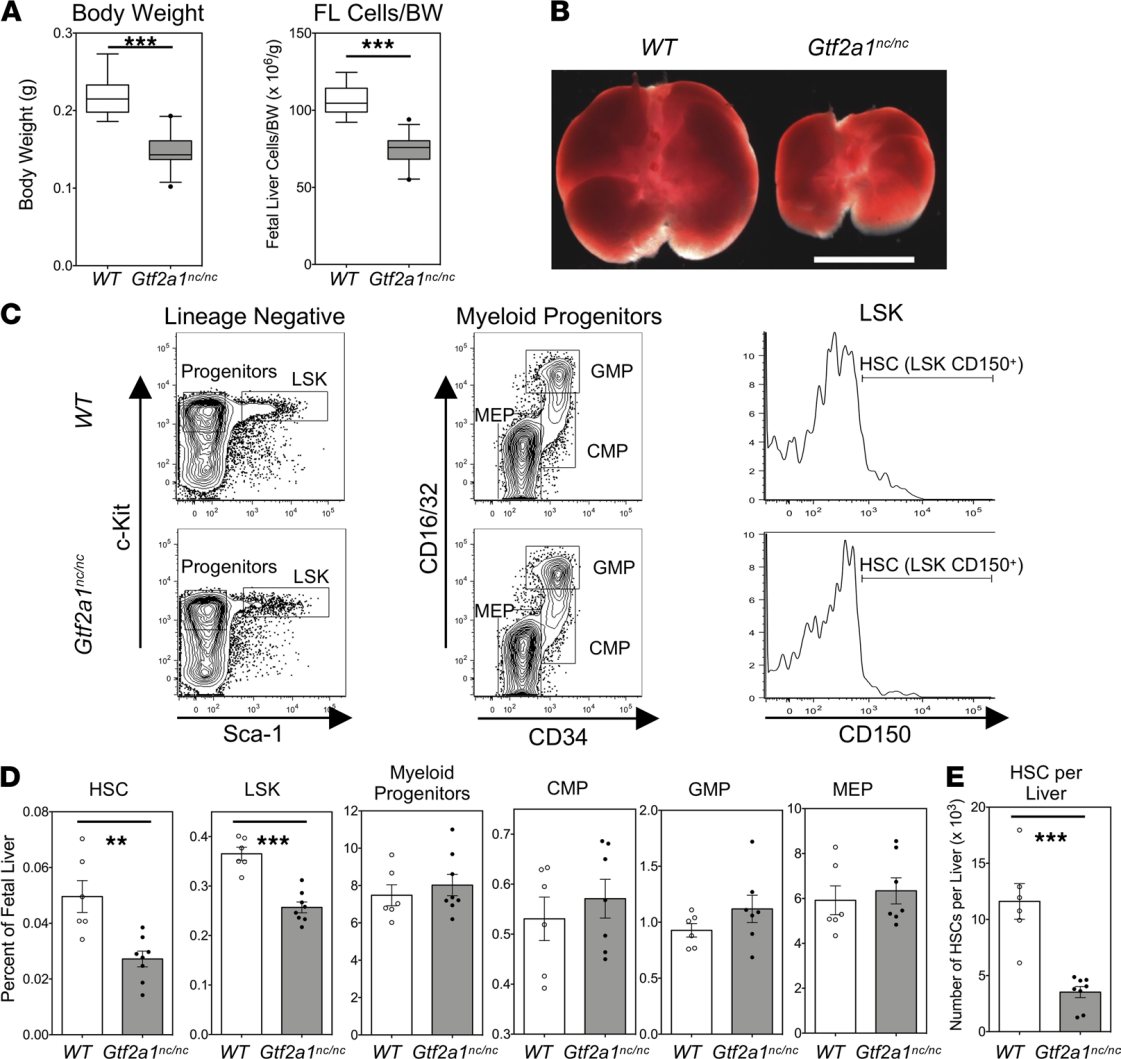
Gtf2a1nc/nc hematopoietic stem cells exhibit overt fetal liver hematopoiesis defects. (**A**) Body weight and normalized fetal liver (FL) cellularity of 19 WT and 27 *Gtf2a1^nc/nc^* E14.5 FLs. Boxes contain the 25th to 75th percentiles of data sets, with 50th percentile center lines, and whiskers mark the 5th and 95th percentiles. Outliers are shown by dots. ***P < 0.001 by Mann-Whitney *U* test. (**B**) Images of E14.5 FLs of the indicated WT and *Gtf2a1^nc/nc^* embryos. Scale bar: 2 mm. (**C** and **D**) Quantification of hematopoietic stem and progenitor cells of E14.5 FLs by flow cytometry. (**C**) Representative dot plots. (**D**) The frequency of stem and progenitor cells in FLs. (**E**) The number of hematopoietic stem cells (HSCs) per FL. Data are shown as the mean ± SEM. **P < 0.01, ***P < 0.001 by Mann-Whitney *U* test (WT, *n* = 6; *Gtf2a1^nc/nc^*, *n* = 8).

**Figure 5:**
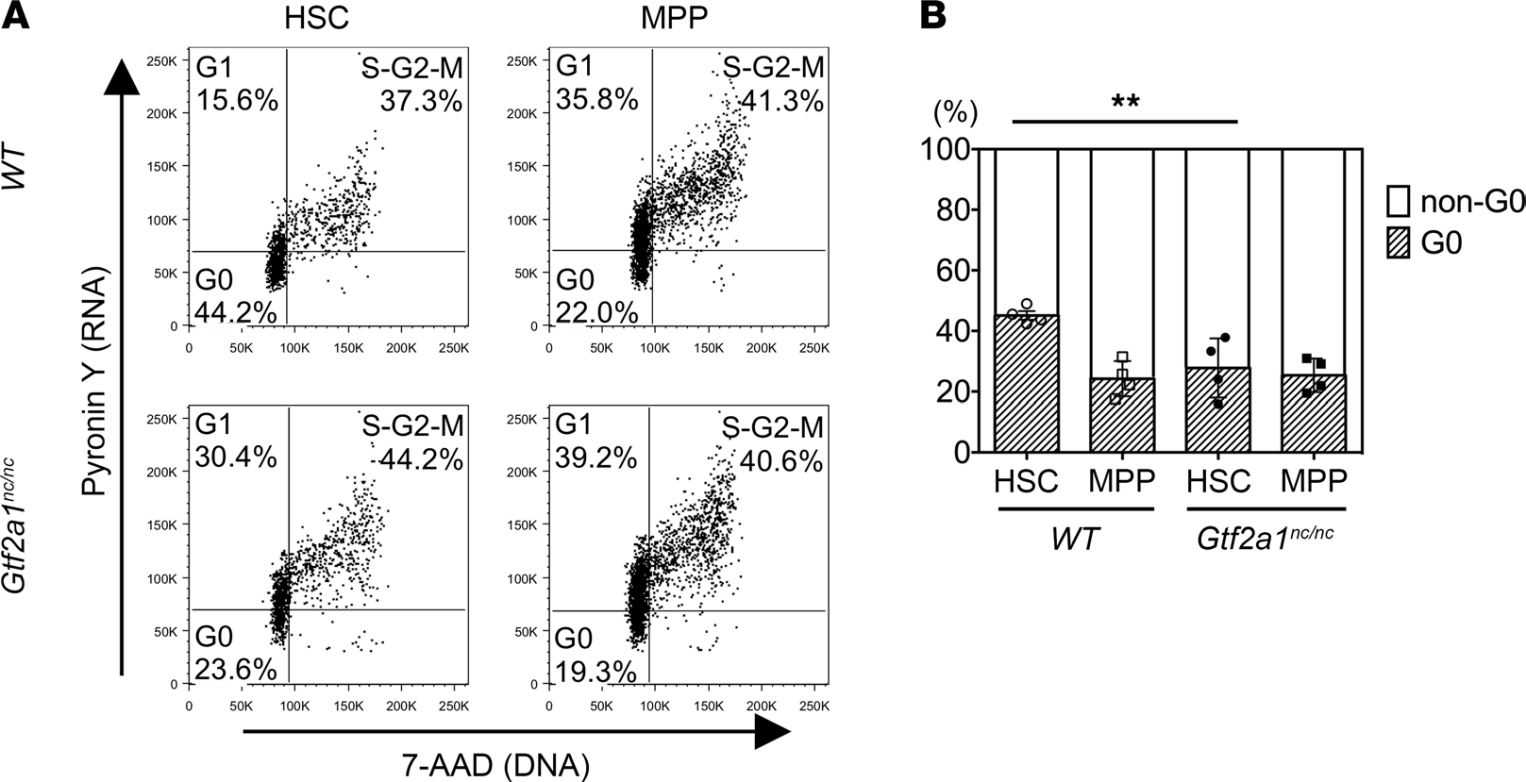
Aberrant cell cycle profile of Gtf2a1nc/nc hematopoietic stem cells. Cell cycle analysis of *Gtf2a1^nc/nc^* E14.5 fetal liver (FL) hematopoietic stem cells (HSC) and multipotent progenitor cells (MPPs). (**A**) The quadrant gates defining G_0_, G_1_, and S/G_2_/M phases. (**B**) The frequency of HSCs and MPPs in G_0_ and non-G_0_ (G_1_ and S/G_2_/M) phases. Data are shown as the mean ± SEM (*n* = 4 for all samples). *P* values of 4 independent experiments by χ^2^ testing were combined by the Fisher’s method; **P < 0.01.

TFIIA family proteins TFIIAα-β and ALF are the only bona fide mammalian Taspase1 substrates known thus far besides MLL1 and MLL2. As expression of ALF is restricted to the mammalian testis (29), we focused on the noncleavage of TFIIAα-β. We have created a *Gtf2a1^nc/nc^* mouse model in which the endogenous D/G cleavage residues of TFIIAα-β are replaced with noncleavable A/A and have reported that *Gtf2a1^nc/nc^* males are infertile (22). Remarkably, *Gtf2a1^nc/nc^* embryos were smaller than their** WT littermates, and their livers were disproportionally smaller (Figure 4, A and B). Furthermore, the frequencies of HSCs in *Gtf2a1^nc/nc^* fetal livers were also reduced (Figure 4D). In fact, the absolute HSC number of *Gtf2a1^nc/nc^* fetal livers was approximately 30% of that of their WT littermates (Figure 4E). Similar to *Tasp1^–/–^* embryos, there were no significant changes in the frequency of myeloid progenitors, CMPs, GMPs, or MEPs in *Gtf2a1^nc/nc^* fetal livers (Figure 4, C and D). Cell cycle analysis of *Gtf2a1^nc/nc^* fetal liver stem and progenitor cells demonstrated decreased a number of *Gtf2a1^nc/nc^* HSCs in the G_0_ phase, which is reminiscent to that of WT and *Gtf2a1^nc/nc^* MPPs (Figure 5). The decrease in G_0_ phase and the coinciding increases in non-G_0_ phases of the *Gtf2a1^nc/nc^* fetal liver HSCs indicated a stem cell quiescence defect similar to that observed in *Tasp1^–/–^* fetal liver HSCs (Figure 2, B and C). These striking similarities between *Gtf2a1^nc/nc^* and *Tasp1^–/–^* fetal livers highlight the importance of the Taspase1-TFIIA axis in the maintenance of fetal liver HSCs.

**Figure 6:**
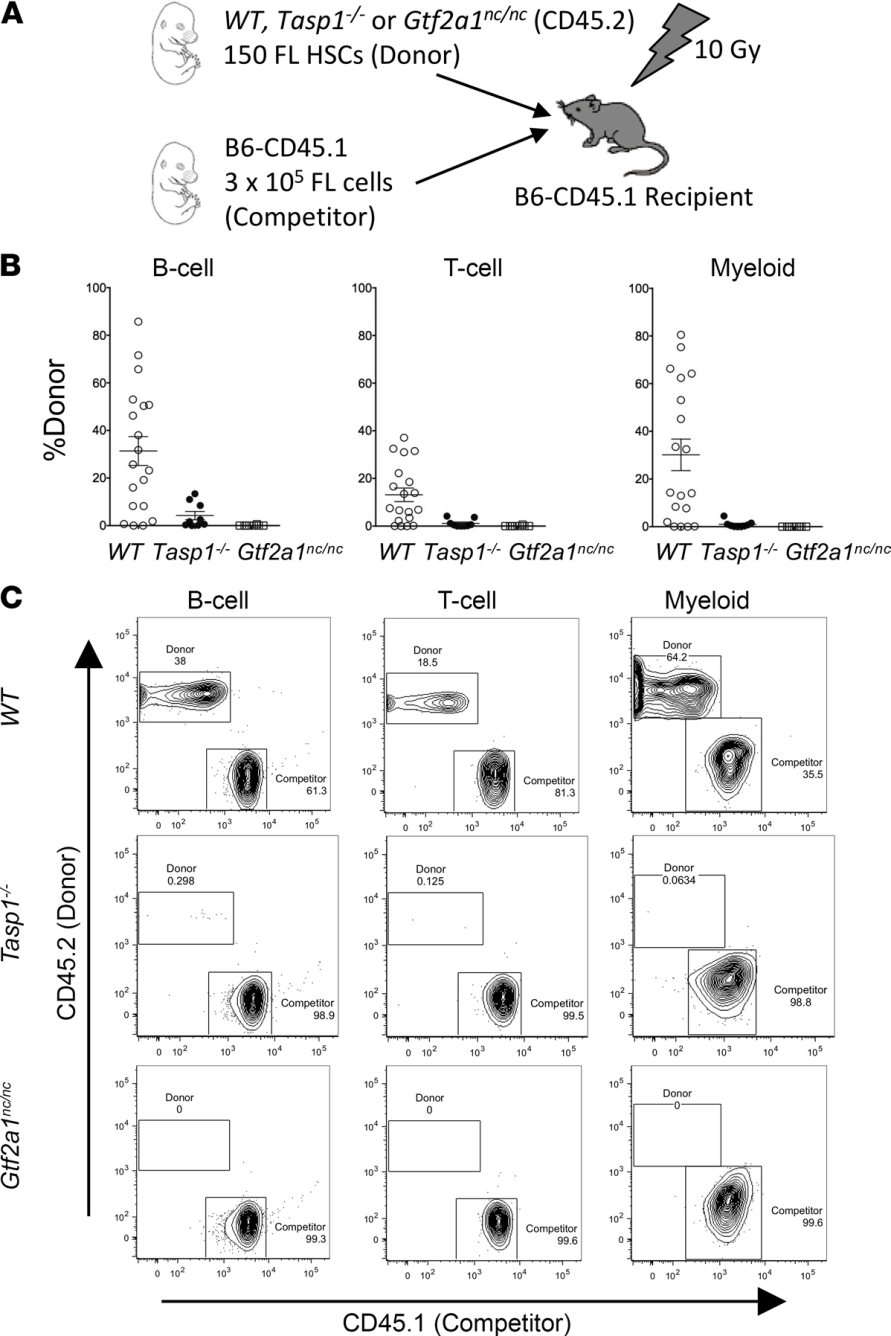
Tasp1–/– and Gtf2a1nc/nc fetal liver hematopoietic stem cells fail to reconstitute lethally irradiated recipient mice. (**A**) Outline of the experimental design of long-term competitive repopulation assays. (**B**) Scatterplots showing the frequency of donor-derived B cell (B220^+^), T cell (CD3^+^) and myeloid (Gr-1^+^Mac-1^+^) lineages reconstituted at 12 weeks after transplant. Data are shown as the average ± SEM. WT, *n* = 19; *Tasp1^–/–^*, *n* = 9; *Gtf2a1^nc/nc^*, *n* = 8. (**C**) Representative plots show reconstitution by donor hematopoietic stem cells (HSCs) of the indicated genotypes in B cell, T cell, and myeloid lineages assessed 12 weeks after transplant. In each lineage, CD45.2^+^ and CD45.1^+^ cells were derived from donor (WT, *Tasp1^–/–^* or *Gtf2a1^nc/nc^*) and competitor cells, respectively. See also Table 1.

To further investigate this potentially novel regulation, we employed stem cell transplant assays to evaluate the capacity of *Tasp1^–/–^* and *Gtf2a1^nc/nc^* fetal liver HSCs in long-term hematopoietic reconstitution. Competitive repopulation assays were performed by transplanting 150 CD45.2^+^ HSCs from WT, *Tasp1^–/–^*, or *Gtf2a1^nc/nc^* E14.5 fetal livers along with CD45.1^+^ competitor cells into lethally irradiated CD45.1^+^ mice (Figure 6A). Twelve weeks after transplantation, peripheral blood was analyzed for the contribution of donor-derived HSCs to mature blood lineages, including B220^+^ for B cells, CD3^+^ for T cells, and Gr-1^+^Mac-1^+^ for myeloid cells (Figure 6, B and C). More than 5% of CD45.2^+^ donor-derived cells in all 3 lineages were detected in 13 of 19 mice transplanted with WT HSCs, whereas multilineage reconstitution was detected in neither the 9 mice transplanted with *Tasp1^–/–^* HSCs nor the 8 mice transplanted with *Gtf2a1^nc/nc^* HSCs (Table 1). Thus, the Taspase1-TFIIA axis appears to be required for long-term reconstitution of hematopoietic cells from fetal livers, suggesting its role in long-term HSC self-renewal.

The phenotypic similarities between *Gtf2a1^nc/nc^* and *Tasp1^–/–^* fetal liver HSCs indicate that TFIIA is the principal Taspase1 substrate conferring Taspase1-orchestrated fetal liver hematopoiesis. As noncleaved TFIIAα-β^nc^ is more stable than cleaved TFIIA (5, 22) and no apparent abnormalities were detected in *Gtf2a1^nc/+^* fetal livers (data not shown), TFIIAα-β^nc^ is unlikely to function as a dominant-negative mutant in fetal liver hematopoiesis. Instead, data favors cleaved TFIIA positively regulating fetal liver hematopoiesis.

**Figure 7:**
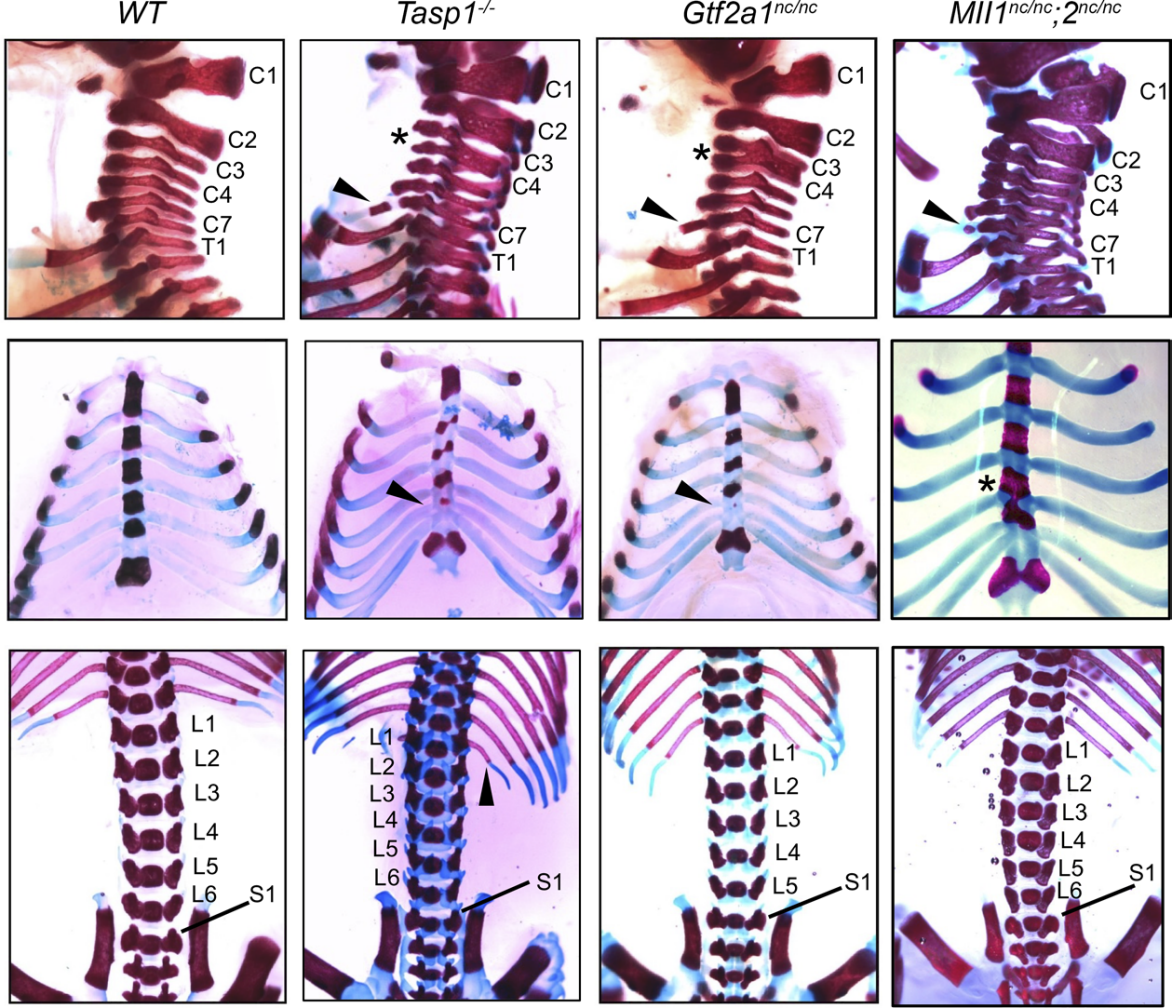
Homeotic transformation of axial skeletons in Tasp1–/–, Gtf2a1nc/nc, and Mll1nc/nc;2nc/nc newborns. Lateral views of cervical (C1–C7) and upper thoracic (T1) regions demonstrate deformed anterior arch of atlas (C1) and fusion of C3–C4 (asterisks) in the *Tasp1^–/–^* and *Gtf2a1^nc/nc^* skeleton and posterior transformation of C7 with an additional rib (arrowheads) in *Tasp1^–/–^*, *Gtf2a1^nc/nc^*, and *Mll1^nc/nc^*;*2^nc/nc^* skeleton (top row). Anterior views of the chest indicate incomplete ossification of sternebrae 4 (arrowheads) in *Tasp1^–/–^* and *Gtf2a1^nc/nc^* newborns and incomplete segmentation of sternebrae 3–4 (asterisks) in *Mll1^nc/nc^;2^nc/nc^* newborns (middle row). Anterior views of lower thoracic and lumbar vertebrae (L1–L6) demonstrate anterior transformation of L1 with an additional rib (arrowhead) in *Tasp1^–/–^* newborns and posterior transformation of L6 in *Gtf2a1^nc/nc^* newborns. See also Table 2.****

*Hox* genes of the vertebrates and homeotic genes of the invertebrates play critical roles in implementing body plan, and their deregulation results in the loss of segmental identities, i.e., homeotic transformation. As our previous studies demonstrated that *Tasp1^–/–^* mice exhibit homeotic transformation (11), we examined the axial skeleton of *Gtf2a1^nc/nc^* newborn pups (*n* = 17) and detected overt homeotic transformations, including abnormal anterior arch of atlas (a.a.a.) (82%), split of C2 (cervical vertebra) (18%), fusion of C3 to C5 (29%), posterior transformation of C7 (65%), anterior transformation of T8 (thoracic) (12%), incomplete ossification of sternebra 4 (65%), incomplete segmentation of sternebrae 3 and 4 (24%), and posterior transformation of L6 (lumbar) (53%) (Figure 7 and Table 2). Unexpectedly, homeotic defects of *Gtf2a1^nc/nc^* newborns were more profound than those of *Mll1^nc/nc^;2^nc/nc^* newborns and highly reminiscent of those of *Tasp1^–/–^* newborns (Figure 7 and Table 2). Homeotic transformation of the axial skeletons is the defining feature of *Hox* gene deregulation (30, 31), and these extensive homeotic defects in *Gtf2a1^nc/nc^* mice indicate a potentially novel regulation of *Hox* genes by TFIIA, a basal transcription factor, through a site-specific proteolytic process.

## Discussion

Unlike most reversible posttranslational protein modifications, such as phosphorylation, acetylation, and methylation, proteolysis through either degradation or site-specific cleavage renders structural changes permanent and, thereby, potentially results in long-lasting functional consequences. In metazoans, site-specific proteolysis regulates critical aspects of biology, such as the activation of blood coagulation factors for hemostasis, the activation of caspases for cell death execution, the cleavage of Notch intracellular domain for cell fate determination, the release of SREBP for cholesterol homeostasis (32), the maturation of HCF and MLL1 for cell cycle progression (11, 12), and the assembly of mature TFIIAα/β/γ for male germ cell maturation (5, 22). Indeed, the identification and functional characterization of proteases and their cognate substrates have been instrumental in unraveling the underlying mechanisms concerning diverse biological processes.

Taspase1 is a highly conserved protease that orchestrates a plethora of genetic programs through cleaving nuclear transcription regulators, MLL1, MLL2, TFIIA, ALF, and dHCF (6, 33). Given that TFIIA is a basal transcription factor and *Hox* genes are highly specialized transcription factors, the connections between Taspase1-mediated proteolytic processing of TFIIA and transcriptional regulation of *Hox* genes were completely unexpected, adding an additional layer of complexity to the intricate control of *Hox* gene expression through upstream transcription factors, epigenetic regulators, and long intergenic noncoding RNA (34, 35) for constructing segmental body plan and specifying cell lineages including stem cells.

The paradigm of Taspase1 function provides an opportunity to investigate how proteases interconnect diverse genetic programs via site-specific proteolysis. In the absence of Taspase1-mediated cleavage, MLL1, MLL2, and TFIIAα-β retain partial activity and animals bearing noncleaved MLL1, MLL2, and/or TFIIAα-β manifest hypomorphism in several biological settings (11, 22, 36, 37). Consequently, the alleles encoding nc MLL1, MLL2, and TFIIAα-β differ from null alleles and thus provide invaluable insight in cases where genetic ablation results in early embryonic lethality. Likewise, Taspase1 deficiency appears to offer a unique opportunity to uncover signaling pathways controlled by developmentally essential genes. Taspase1 regulates the cell cycle, axial skeletal formation, fetal HSC homeostasis, male germ cell development (11, 22, 36, 37), and craniofacial development (23). As TFIIA complexes with not only TBP but also TRF2 (a male germ cell–enriched TBP variant) and TRF3 (a vertebrate-specific TBP variant), the Taspase1-TFIIA axis is positioned to orchestrate the assembly of diverse tissue-specific or context-dependent transcription machinery that are crucial for organismal development.

## Methods

### Mice.

*Tasp1^–/–^*, *Mll1^nc/nc^*, *Mll2^nc/nc^*, and *Gtf2a1^nc/nc^* mice have been previously described (11, 22). *Tasp1^+/–^* and *Gtf2a1^nc/+^* mice were backcrossed to the WT C57BL6/J strain for 6 generations. *Mll1^nc/nc^* and *Mll2^nc/nc^* mice were similarly backcrossed to the WT C57BL6/J strain for 10 generations.

### Flow cytometric analyses of hematopoietic cells.

To obtain single-cell suspensions of hematopoietic cells, fetal livers were removed from E14.5 mouse embryos and homogenized by 3 passages through a 21-gauge needle. Freed cells were filtered through a nylon mesh with a pore size of 40 μm. Collected cells were treated with Red Blood Cell Lysing Buffer (MilliporeSigma) and then washed and filtered through nylon mesh. Automated blood cell counting was performed using a Hemavet Blood Analyzer (Drew Scientific). For mature blood cell analyses, 1 million cells were stained using the following antibodies: B220 APC (RA3-6B2) (BD Biosciences), Gr-1 PE (RB6-8C5) (BD Biosciences), and TER-119 Pacific Blue (TER-119) (BioLegend). For analyses of stem and progenitor cells, 1 million cells were stained using a combination of following antibodies: Lin (lineage antibody cocktail from BioLegend: CD3 [17A2], CD4 [GK1.5], CD8α [53-6.7], B220 [RA3-6B2], TER-119 [TER-119], and Gr-1 [RB6-8C5]) Pacific Blue (BioLegend), Sca-1 PE (D7) (BD Biosciences), c-Kit APC/Alexa Fluor 750 (2B8) (BD Biosciences), CD34 FITC (RAM34) (BD Biosciences), CD16/32 Alexa Fluor 647 (clone 93) (eBioscience), and CD150 PE/Cy7 (TC15-12F12.2) (BioLegend). To identify and exclude dead cells, 7-AAD was added in the final suspension. Cells positive for Sca-1, CD34, or CD150 were determined by fluorescence-minus-one controls. Cells were analyzed with an LSRFortessa flow cytometer (BD Biosciences). Dot plots and histograms were made with FlowJo software. Graphs and statistical analyses were prepared with GraphPad Prism 6.

### Sorting of hematopoietic stem and progenitor cells.

Single-cell suspensions were prepared from fetal livers of E14.5 embryos or adult bone marrow and stained as described above. Fetal liver cells from multiple littermate embryos of the same genotype were pooled to ensure sufficient number of cells for staining. Stained cells were subsequently double sorted using a FACSAria II (BD Biosciences) to a final purity of more than 95%.

### Competitive reconstitution assay.

C57BL6/J-CD45.1 mice were used as recipients and competitors. The recipient mice were irradiated by 10 Gy divided into 2 fractions on the day –1. Competitor cells were prepared from E14.5 fetal livers from the C57BL6/J-CD45.1 mice and frozen in advance. On day 0, LSK CD150^+^ HSCs were sorted from E14.5 fetal livers of donor embryos that express CD45.2. Each recipient mouse was transplanted with 150 donor HSCs and 3 *×* 10^5^ competitor fetal liver cells by tail vein injection. After 12 weeks, peripheral blood and bone marrow cells of recipients were analyzed for the presence of CD45.1^+^ and CD45.2^+^ cells by a LSRFortessa. Peripheral blood mononucleic cells were stained with the following antibodies for analyzing myeloid lineage: CD45.1 PE (A20) (BD Biosciences), CD45.2 FITC (clone 104) (BD Biosciences), Mac-1 Ax647 (M1/70) (BD Biosciences), and Gr-1 Pacific Blue (RB6-8C5) (BioLegend). The following antibodies were used for lymphoid lineage: CD45.1 PE (A20), CD45.2 FITC (clone 104), B220 APC (RA3-6B2), and CD3 Pacific Blue (17A2).

### Cell cycle analysis.

Suspensions of sorted hematopoietic stem and progenitor cells were fixed with ethanol overnight at 4°C, washed, and then resuspended in the house-made Nucleic Acid Staining Solution (38). Cells were stained with 7-AAD at room temperature for 20 minutes, followed by pyronin Y staining on ice for 15 minutes. Stained cells were analyzed by a LSRFortessa flow cytometer.

### Annexin V assay.

One million fetal liver cells were stained with the following antibodies: Lin Pacific Blue, Sca-1 PE (D7), c-Kit APC/Ax750 (2B8), and CD150 PE/Cy7 (TC15-12F12.2). The cells were subsequently washed, stained with annexin V FITC (BioVision) and Propidium Iodide (MilliporeSigma), and then analyzed by flow cytometry.

### Skeletal studies.

P1 newborns were sacrificed and stained using Alizarin red (MilliporeSigma) and Alcian blue (MilliporeSigma) for bone and cartilage, respectively, as described previously (39).

### Statistics.

Statistical significance was evaluated using the Mann-Whitney *U* test for continuous variables, unless otherwise specified. χ^2^ testing was performed on cell cycle analyses, and *P* values of independent experiments were combined by the Fisher’s method. *P* values of less than 0.05 were considered significant.

### Study approval.

All animal work was performed in accordance to a protocol approved by the Institutional Animal Care and Use Committee of Memorial Sloan Kettering Cancer Center.

## Author contributions

JJH designed the study. HN, ACS, and ST performed experiments and acquired and analyzed data. SAA, CYP, EHC, and JJH supervised experiments and analysis. HN and JJH wrote the manuscript.

## Figures and Tables

**Table 2:**
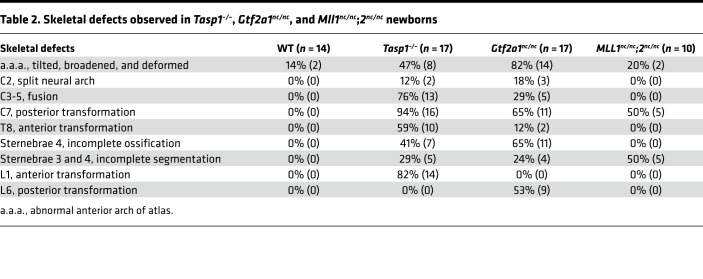
Skeletal defects observed in *Tasp1^–/–^*, *Gtf2a1^nc/nc^*, and *Mll1^nc/nc^*;*2^nc/nc^* newborns

**Table 1:**
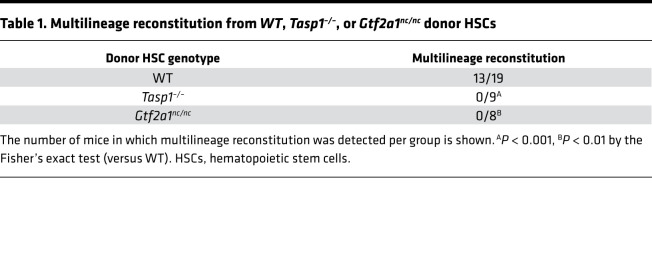
Multilineage reconstitution from *WT*, *Tasp1^–/–^*, or *Gtf2a1^nc/nc^* donor HSCs

## Version 1 06/22/2021

In-Press Preview

## Version 2 08/09/2021

Electronic publication
